# Case Report: PET-CT ischemia and viability maps guiding an emergency revascularization

**DOI:** 10.3389/fcvm.2025.1637403

**Published:** 2025-10-29

**Authors:** Dionysios Adamopoulos, Léo Meyer de Stadelhofen, François Mach, Valentina Garibotto, Stéphane Noble

**Affiliations:** ^1^ Department of Medical Specialties, Cardiology, Geneva University Hospital, Geneva, Switzerland; ^2^ Faculty of Medicine, University of Geneva, Geneva, Switzerland; ^3^ Diagnostics Department, Division of Nuclear Medicine and Molecular Imaging, Geneva University Hospital, Geneva, Switzerland; ^4^ Department of Medical Specialties, Primary Care Division, Geneva University Hospital, Geneva, Switzerland

**Keywords:** nuclear cardiac imaging, coronary artery disease, myocardial viability, CTO—percutaneous coronary intervention, myocardial ischemia

## Abstract

A 60-year-old woman was scheduled for elective coronary angiography after a positron emission tomography and computer tomography (PET-CT) cardiac perfusion imaging test showing extensive myocardial ischemia. A few hours before the scheduled angiography, she presented to the emergency room with chest pain and diffuse ST-segment modifications leading to emergent coronary angiography. Recent PET-CT ischemia and viability maps were available at the time of the intervention, favoring a percutaneous coronary intervention for chronic total occlusion (CTO PCI) of the left circumflex artery, apart from the culprit lesion of the left main coronary artery and left anterior descending artery, with good results. A second PET-CT scan 23 days post-PCI showed a reversible perfusion defect of the first diagonal branch territory, which was subsequently treated. A rapid normalization of the left ventricular ejection fraction (LVEF) was noted after revascularization, while the second PET-CT showed no signs of significant myocardial necrosis. This case illustrates the potential role of cardiac imaging perfusion studies in guiding revascularization in complex cases in the context of an acute myocardial infarction (MI).

## Learning objectives

• Identify and interpret myocardial perfusion defects by nuclear cardiac imaging, in conjunction with coronary lesions to guide decisions for revascularization.

• Understand the potential role and prognostic significance of myocardial viability in CTO PCI for selected, complex cases.

A 60-year-old woman was referred to our cardiology clinic for chest pain evaluation. The patient reported brief episodes of chest pain on exertion, which began approximately 6 months earlier. The pain is located centrally and radiates to both shoulders. The intensity was variable and appeared principally on exertion, but also after meals. No other symptoms were reported. The physical examination was unremarkable with no signs of heart failure or peripheral vascular disease, while vital signs were in the normal range.

## Past medical history

Her medical history was free from prior cardiovascular (CV) events and included substituted hypothyroidism and gastric bypass intervention. The patient presented multiple CV risk factors (type 2 diabetes, arterial hypertension, and dyslipidemia), all well controlled under optimal medical therapy. She led a sedentary life and was overweight (BMI 28.4 kg/m^2^).

## Investigations

**Figure 1:**
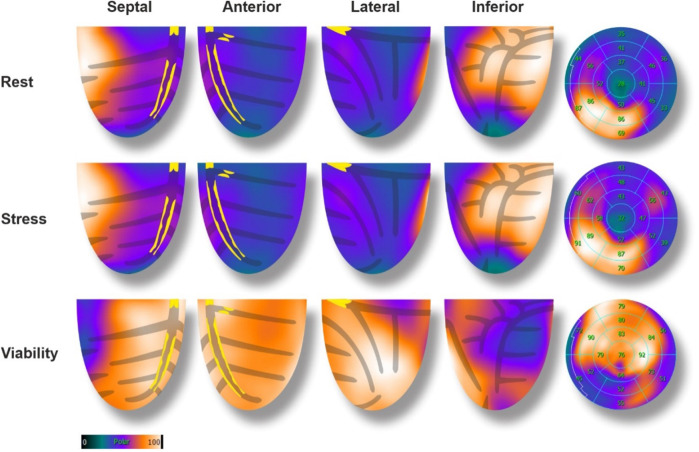
Baseline 82-rubidium PET-CT scan with corresponding glucose metabolism (18F-FDG), and coronary lesions. In perfusion images, a large (70%), severe, anterior, lateral, and apical defect was noted with no reversibility. The 18F-FDG showed enhanced glucose metabolism in the hypo-perfused areas (mirror image), denoting a hibernating state of the myocardial cells.

A gastroscopy was performed a few days before the cardiologist's visit, showing signs of esophagitis, and a high-dose proton pump inhibitor treatment was initiated. Due to the intermediate-high baseline CV risk and the presence of symptoms, non-invasive testing for myocardial ischemia was scheduled (PET-CT scan). The exam was realized using a Siemens BIOGRAPH VISION® camera, with injection of the 82-Rubidium tracer (600MBq per phase). Stress coronary vasodilation was attained pharmacologically by a selective A2A adenosine receptor agonist (Regadenoson). A CT of the thorax was also obtained concomitantly before the PET image acquisitions, for the application of the attenuation correction algorithm and the estimation of the coronary calcium score. The typical PET-CT scan protocol includes semiquantitative and quantitative left ventricle (LV) perfusion evaluation as well as LV volume measures during the cardiac cycle [LV ejection fraction (EF) estimation]. The exam revealed a massive (70% of the LV) and severe perfusion defect of the anterior, lateral, apical, and septal walls of the LV, both at rest and during stress, with no reversibility, highly suggestive of transmural necrosis (Figure 1). Myocardial blood flow quantification showed a severe decrease in flow and coronary flow reserve (CFR) in the same territories. A transient dilation of the LV was noted, and LVEF was decreased at rest (47%) and even more during stress (40%). The coronary artery calcium score was measured at 2449 Agatston units (AU), denoting extensive coronary artery disease.

Two days later, a viability PET-CT exam was performed using the ^18^F-fluorodeoxyglucose (18F-FDG) tracer (231 MBq) after administration of glucose, 500 mg of Acipimox, and insulin according to standard procedures (1). The exam showed preserved metabolic activity of the LV perfusion defect areas, denoting preserved viability of the anterior, lateral, apical, and septal walls of the LV (hibernation, Figure 1). A coronary angiography was scheduled for the next day.

## Management

The same night after dinner, the patient felt the same high-intensity chest pain radiating to both shoulders, which led her to the emergency room. The initial physical examination revealed fine crackles on the lung bases, which rapidly evolved into pulmonary edema and hemodynamic instability. The electrocardiogram (ECG) showed signs of diffuse ischemia with a profound ST-segment depression in DII, DIII, aVF, and V4–V6, with concomitant ST-segment elevation in the aVR derivation. Blood tests revealed a positive high-sensitivity troponin (1,438 ng/L), increased creatinine kinase (CK) (505 U/L), and increased pro-brain natriuretic peptide values (6,775 ng/L).

The patient was sent directly to the catheterization laboratory for coronary angiography in the context of ST depression in more than 6 leads and ST elevation in aVR suggestive of severe multivessel ischemia or left main (LM) coronary disease.

**Figure 2:**
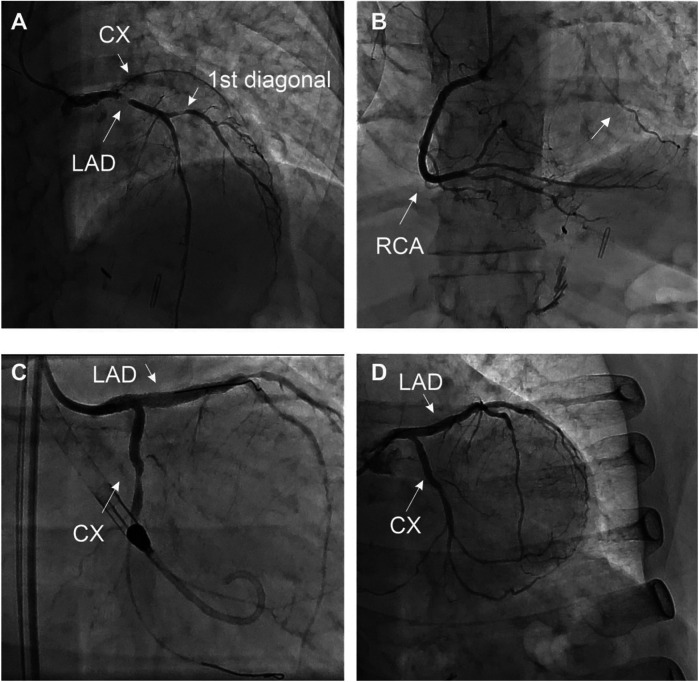
Coronary angiography at the initial presentation **(A–C)** and final result **(D)** the patient presented with extended 2-vessel coronary artery disease with a 50%–70% lesion of the distal LM, a sub-occlusive lesion of the proximal LAD with diffuse atherosclerotic infiltration of the median and distal part, à 70% stenosis of the 1st diagonal, and a CTO of the LCX collateralized by the RCA (arrow). MT, LAD, and LCX were initially treated by PCI under mechanical circulatory support (Impella CP system®). The 1st diagonal was treated during the second angiography **(D)**, after documentation of the residual ischemia by PET-CT (Figure 4).

The exam revealed a severe 2-vessel coronary artery disease with a 50%–70% stenosis of the distal LM, sub-occlusive stenosis of the proximal left anterior descending artery (LAD) with diffuse atherosclerosis of the median and distal part, a 70% stenosis of the 1st diagonal artery and total occlusion of the proximal left circumflex artery (LCX) receiving collaterals from the right coronary artery (RCA) (Figure 2).

Due to the hemodynamic instability, a mechanical circulatory support was inserted (Impella CP System®, Abiomed) at the beginning of the procedure via femoral access. The procedure included percutaneous coronary intervention (PCI) of the LM and proximal LAD using Shockwave intravascular lithotripsy as well as recanalization of the proximal LCX artery using a Fileder XTA 0.14 wire (Asahi Intecc, JN) to cross the lesion and a T-stenting strategy (Medtronic Onyx stent 2.75 × 22 mm in the LCX with post-dilatation at 3.0 mm, 3.5 × 30 mm Onyx stent in the LAD and LM with proximal optimization à 4.0 mm). No significant complications were noted. The patient was transferred to the intensive care unit (ICU), under double anti-aggregation treatment by Aspirin® and Prasugrel® at standard doses.

The patient rapidly improved with a quick hemodynamic stabilization, leading to the ablation of the Impella CP System® a few hours later. The signs of left heart failure disappeared under diuretics. Transthoracic echocardiography (TTE) at the ICU showed an LVEF of 40% immediately after the ablation of the Impella CP System®. The maximum high sensitivity troponin was measured at 12,408 ng/L, and CK reached 3,706 U/L. No significant arrhythmias were noted, and the patient was discharged from the ICU two days later.

**Figure 3:**
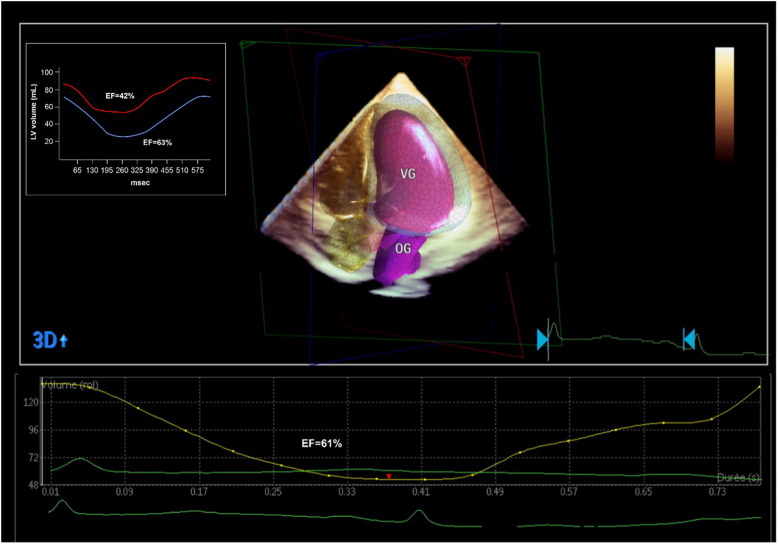
Echocardiographic and PET-CT evaluation of the LVEF after the coronary revascularization. PET-CT showed a significant improvement in LVEF (from 42% to 63%) with a significant decrease globally in LV volumes, denoting an ischemic LV dilatation. 3D echocardiographic evaluation confirmed the normalization of the LVEF.

A TTE at day 5 showed complete recovery of the LVEF without any regional abnormalities (Figure 3).

**Figure 4:**
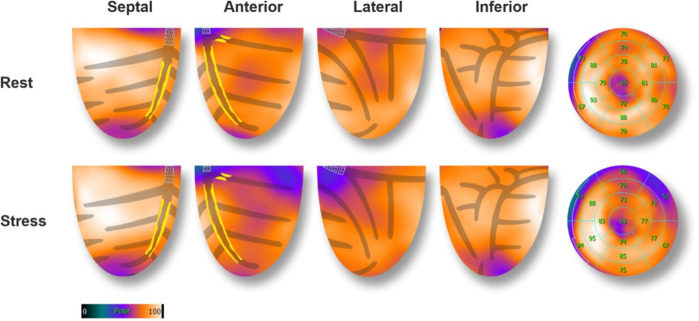
82-Rubidium PET-CT scan 23 days after the PCI of the MT, proximal LAD, and CX arteries. Compared to the baseline exam, images at rest showed a significant improvement in myocardial perfusion after the revascularization. Images during pharmacological stress showed an 8% severe defect in perfusion of the anterolateral basal segment, corresponding to the territory of the 1st diagonal artery (70% focal stenosis of the proximal part). A PCI was subsequently performed (Figure 3).

A second 82-Rubidium PET-CT scan 23 days post-PCI showed significant improvement in myocardial perfusion at rest (4% apical) with a severe but completely reversible perfusion defect of the basal, anterolateral segment of the LV, suggesting a 7%–8% residual ischemia in the area of the 1st diagonal stenosis (Figure 4). The LVEF was significantly increased both at rest (57%) and during pharmacological stress (62%). Subsequently, a PCI of the 1st diagonal branch was performed without complications (2.5 × 12 mm Onyx stent). A control coronary angiography at 18 months showed persistent good results of the LM, LAD, and LCX PCI.

## Discussion

Myocardial ischemia is a key factor for guiding revascularization strategy in patients with stable coronary artery disease (2). The role of myocardial viability, though, is less clear. Historically, many observational studies and meta-analyses have suggested that in patients with dysfunctional myocardium, revascularization is associated with better outcomes and improved ventricular recovery when myocardial viability has been proven by advanced imaging (3). However, more recent controlled trials (STICH and REVIVED-BCI2) showed no benefit in survival with revascularization compared to optimal medical treatment in patients with decreased LVEF despite preserved myocardial viability (4, 5). These findings do not apply in acute, complex cases where viability may help differentiate hibernating myocardium from scar. This is extremely relevant for predicting segmental functional recovery after revascularization in the acute setting, even if this does not translate into improved clinical outcomes in the general population (6). However, this information is rarely available in the context of acute coronary syndromes. In this case, the patient presented to the emergency room with chest pain and ECG signs of diffuse myocardial ischemia. The urgent coronary angiography confirmed the presence of a severe 2-vessel disease involving the distal LM, proximal LAD, and LCX arteries. This was suspected after the initial 82-Rubidium PET-CT scan showing a massive, severe non-reversible perfusion defect covering the anterior, lateral, septal, and apical walls of the left ventricle (Figure 1). Although the LAD lesion was the culprit lesion for the acute coronary syndrome and ECG manifestations of ischemia, the LCX artery was found occluded with morphological aspects pointing to chronic total occlusion (CTO). Typically, in this case without information on the viability of the lateral wall, a recanalization of the LCX artery may not have been attempted, considering the technical challenge. However, the readily available viability test encouraged the operator to attempt the recanalization of the occluded LCX artery in an attempt to prevent further myocardial damage (7). The 18F-FDG viability test performed a few hours before the angiography, confirmed the presence of enhanced glucose metabolism in this area, which denotes a preserved viability of the myocardial cells, thus pointing to the presence of hibernating myocardium and not a scar. This information favored the prompt attempt for the recanalization of the LCX CTO at the time of the treatment of the culprit lesion (LM-LAD). This decision possibly explains the excellent short and mid-term results of the intervention, with the very rapid (in a few hours) restoration of the hemodynamic stability (ablation of the Impella CP system®), the normalization of the LVEF (62% vs. 40%, Figure 3) and the absence of significant myocardial defect at the latest perfusion PET-CT scan (Figure 4).

Although many studies have assessed the role of viability testing in stable coronary artery disease, this case is unique in that myocardial perfusion and viability information were readily available in the context of an acute coronary syndrome. It also reinforces the evidence that, in selected cases, viability assessment may be important for guiding revascularization—especially when performed early after coronary occlusion.

However, it is technically and logistically challenging to implement viability or even perfusion testing during an acute coronary syndrome. These tests often require the administration of vasoactive drugs, which may compromise hemodynamic stability. Additionally, both perfusion and 18F-FDG testing require preparation that can delay angiography, which remains the cornerstone of treatment in this setting.

## Conclusion

The present case is an excellent reminder of the importance of early revascularization in achieving favorable results in acute coronary syndromes. It also underscores that, in selected complex cases, viability testing in conjunction with perfusion imaging can play a critical role in guiding revascularization strategy and predicting outcomes.

## Data Availability

The datasets presented in this study can be found in online repositories. The names of the repository/repositories and accession number(s) can be found in the article/Supplementary Material.
